# Identification of the DNA Repair Defects in a Case of Dubowitz Syndrome

**DOI:** 10.1371/journal.pone.0054389

**Published:** 2013-01-25

**Authors:** Jingyin Yue, Huimei Lu, Shijie Lan, Jingmei Liu, Mark N. Stein, Bruce G. Haffty, Zhiyuan Shen

**Affiliations:** The Cancer Institute of New Jersey, Robert Wood Johnson Medical School, New Brunswick, New Jersey, United States of America; University Medical Center Hamburg-Eppendorf, Germany

## Abstract

Dubowitz Syndrome is an autosomal recessive disorder with a unique set of clinical features including microcephaly and susceptibility to tumor formation. Although more than 140 cases of Dubowitz syndrome have been reported since 1965, the genetic defects of this disease has not been identified. In this study, we systematically analyzed the DNA damage response and repair capability of fibroblasts established from a Dubowitz Syndrome patient. Dubowitz syndrome fibroblasts are hypersensitive to ionizing radiation, bleomycin, and doxorubicin. However, they have relatively normal sensitivities to mitomycin-C, cisplatin, and camptothecin. Dubowitz syndrome fibroblasts also have normal DNA damage signaling and cell cycle checkpoint activations after DNA damage. These data implicate a defect in repair of DNA double strand break (DSB) likely due to defective non-homologous end joining (NHEJ). We further sequenced several genes involved in NHEJ, and identified a pair of novel compound mutations in the DNA Ligase IV gene. Furthermore, expression of wild type DNA ligase IV completely complement the DNA repair defects in Dubowitz syndrome fibroblasts, suggesting that the DNA ligase IV mutation is solely responsible for the DNA repair defects. These data suggests that at least subset of Dubowitz syndrome can be attributed to DNA ligase IV mutations.

## Introduction

Dubowitz Syndrome, firstly reported in 1965, is an autosomal recessive disorder with a unique set of clinical features, including microcephaly, short statures, mild mental retardation, eczema, distinct facial features, etc. [1]–[5] The genetic base of Dubowitz syndrome has not been elucidated, although chromosome 13q micro-deletion or duplication has been reported [6]. Some Dubowitz syndrome patients have elevated chromosome breakages and tumor formations [7]–[11], implying potential defects in DNA repair. While certain Dubowitz syndrome cases have abnormal production of immunoglobulins, but many other cases did not display immunoglobulin deficiency at young ages [7], [10], [12]. Bone marrow failures have been reported in a few cases of Dubowitz syndrome [13], [14]. These clinical phenotypes overlap with some of the DNA repair deficient syndromes such as Nijmegen Breakage Syndrome (NBS) syndrome, Fanconi anemia, DNA ligase IV syndrome, Bloom's syndrome, etc. But direct evidence for DNA repair defects in Dubowitz syndrome has not been reported.

In this study, we report the cellular and genetic characterization of a Dubowitz syndrome patient who was diagnosed in 1973 [3], and developed anal cancer later. We established a fibroblast cell line, and we found that the fibroblasts are hypersensitive to ionizing radiation and several other DNA damage agents, due to the defect in the repair of DNA double strand break (DSB) as a result of DNA Ligase IV mutations. We suggest that defective DNA repair contributes to development of at least a subset of Dubowitz Syndrome, offering a molecular base for this poorly understood inheritable disease. This new finding will be valuable to guide treatment selection for Dubowitz Syndrome patients with cancer.

## Results

### Identification of a radiation sensitive patient with Dubowitz syndrome

**Figure 1:**
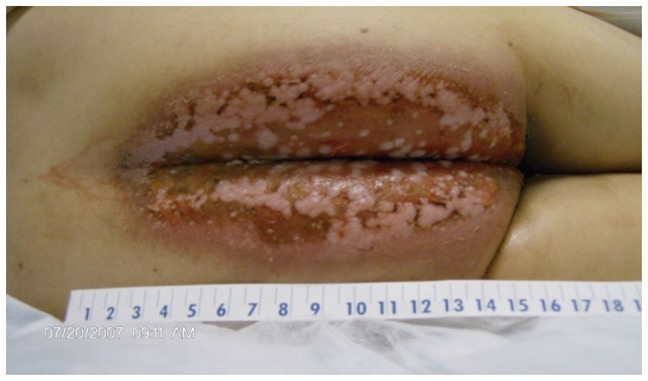
Severe skin reaction of a Dubowitz Syndrome patient to radiation therapy. Shown is the severe skin injury on the anal-rectal region after 10 fractions of 2 Gy radiation-therapy for anal cancer. The patient died of the metastatic anal cancer later.

A one-year old female patient was diagnosed with Dubowitz syndrome in 1973 by Optiz et al. due to typical clinical features of Dubowitz syndrome [3], [15]. At age of 34 years, she presented with bright red blood per rectum. Colonoscopy revealed a 5 cm ulcerative mass located at the posterior rectum just inside the anal verge. Biopsy was positive for squamous cell carcinoma. Due to her multiple medical issues, including low platelets, low white count, anemia, the patient was not felt to be a candidate for surgery or chemotherapy. After discussion with the family and other members of the multidisciplinary medical team it was decided to proceed with limited field radiation therapy alone directed at the lower rectum and anal region. After only 10 treatments of 200 cGy fractions per day to a small anterior and posterior radiation field directed at the anal/lower rectal region, the patient developed severe moist desquamation in the per-rectal, vaginal and pubic region. Over the ensuing several weeks the patient severe moist desquamation gradually healed, with significant residual induration and fibrosis in the region (Figure 1). There was approximately a 50% regression of the tumor by physical examination during this time. The patient died of widespread cancer metastasis and failed local cancer control.

### Establishment of a primary fibroblast cell line for the Dubowitz syndrome

**Figure 2:**
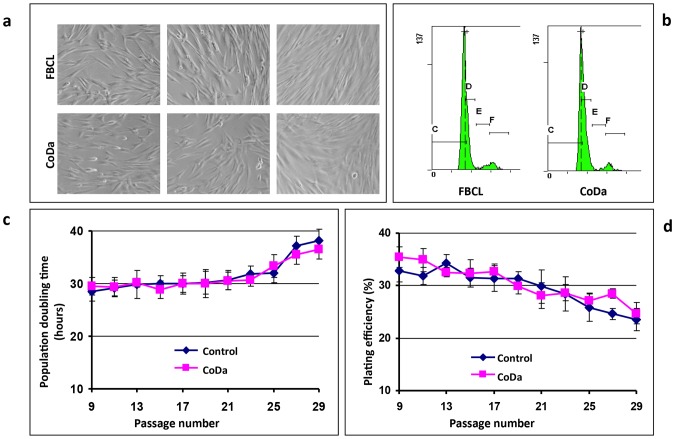
Primary culture of human fibroblasts. Fibroblast cell lines, designated CoDa, were established from the autopsy skin tissues. Panel a shows the representative images of a normal fibroblast control line (FBCL) and CoDa cells in *in vitro* culture. Images were taken under bright field microscopy with various confluences. Panel b shows the cell cycle distribution of fibroblasts in log-phase of in vitro culture, based on flow cytometry analysis of the DNA content. Panels c and d show population doubling time (panel c) and plating efficiency (panel d) of CoDa and control fibroblasts *in vitro*. Shown are averages of 2–3 independent experiments with CoDa-33 and FBCL cells. Error bars are standard errors.

To facilitate the identification of the genetic defects of Dubowitz syndrome, we established primary dermal fibroblasts, designated CoDa from autopsy skin tissues (see Materials and Methods for procedures of establishing the primary couture). Early passages of CoDa cells were used for growth property analysis along with a normal primary fibroblast control line FBCL as the control. The CoDa cells displayed typical fibroblast morphology in primary culture, and have comparable growth properties and cell cycle distribution as normal skin fibroblasts at log-phase of growth (Figs. 2a and 2b).

As shown in Figure 2c, both cells have population doubling time of ∼30 hours. The CoDa cells maintain stable growth rate for up to passage 25 in continuous *in vitro* culture, similar as control fibroblast FBCL cells. As shown in Figure 2d, the plating efficiency (PE) for both cells is around 30–34% at early passages, and gradually decreases as the passage numbers increase. Collectively, Figure 2 suggests that the CoDa cells have similar growth properties as the normal control cell line.

### Radiation sensitivity of the Dubowitz syndrome fibroblasts

**Figure 3:**
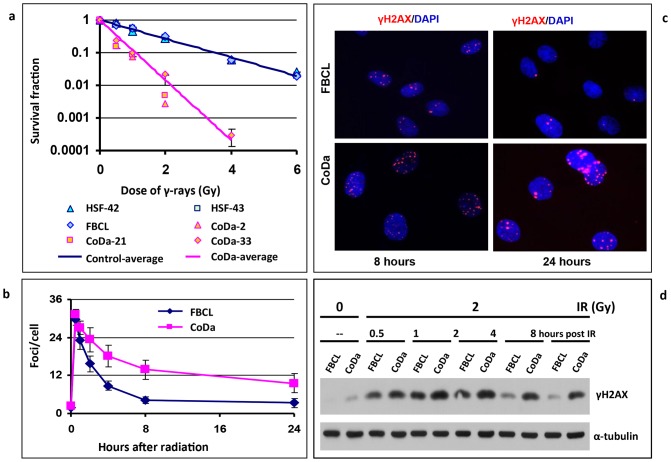
CoDa cells are hyper-sensitive to ionizing radiation and have defects in DNA double-strand break repair. (a) Cellular sensitivity of human fibroblasts to Cs-137 γ-radiation measured by colony formation assay. Shown are the averages of 3–6 experiments with three CoDa clones (CoDa-2, 21, and 33), and 3–7 experiments with three independent control fibroblasts cell lines (FBCL, HSF-42, and HSF-43). Error bars are standard errors. (b) The numbers of γH2AX foci per cell in a normal fibroblast control line (FBCL) and CoDa cells. At different time points after the cells were treated with γ-radiation, cells were fixed and stained by immunoflurescent techniques with an anti-γH2AX antibody. The number of γH2AX foci was counted in >400 cells per time point in each experiment. Shown are averages and standard deviations of three experiments. (c) The representative images of γH2AX nuclear foci at 4 and 24 hours after IR treatment. DAPI staining shows the location of nuclear. (d) The total γH2AX protein level measured by anti-γH2AX western blot after irradiation (2 Gy) in FBCL and CoDa cells. β-actin was used as a loading control.

To confirm the radiation sensitivity of the CoDa cells, we performed colony formation assay. As shown in Figure 3a, the CoDa cells are ∼30 fold more sensitive to ionizing radiation than normal fibroblasts at the dose of 2 Gy (0.99% survival of CoDa cells vs. 29.7% survival for normal fibroblasts). To achieve 10% of survival fraction, it takes 3.49 Gy of γ-irradiation for normal fibroblasts, but it takes only 1.08 Gy for CoDa cells. Because the repair of DNA double strand breaks (DSB) is the most dominant determinant affecting cell sensitivity to ionizing radiation, and phosphorylated histone H2AX (γH2AX) is a reliable surrogate marker for DSB [16]–[19], we measured the kinetics of γH2AX nuclear foci following 2 Gy of γ-irradiation. The number of foci per cell increased to a similar level in CoDa and control cells shortly after 2 Gy irradiation (Fig. 3b, c), suggesting that irradiation induced a similar level of initial amount of DNA damage in these cells. However, there is a significant delay in the clearance of γH2AX foci among CoDa cells than the control fibroblasts. The same extend of initial activation but delayed clearance of γH2AX was confirmed by western blot after 2 Gy of irradiation (Fig. 3d). By 24 hours after irradiation, there was still a significant level of γH2AX in CoDa cells, while the intensity of γH2AX signal in the control cells returned to the basal level.

**Figure 4:**
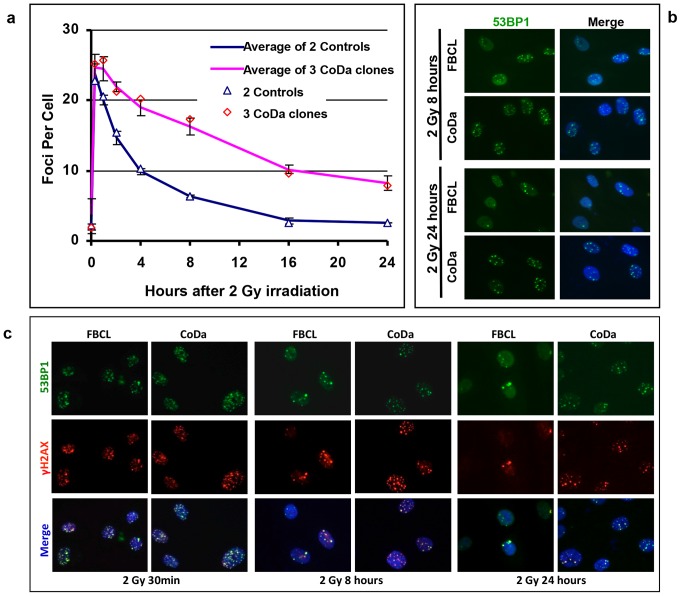
CoDa cells have normal IR-induced formations of 53BP1 nuclear foci, but displayed clearance of 53BP1 foci. The number of nuclear 53BP1 foci was scored at various time points after 2 Gy of γ-irradiation in two control fibroblasts (FBCL and HSF42) and three CoDa populations (CoDa-2, CoDa-21, and CoDa-33). (a) The average number of 53BP1 foci from control fibroblasts (Δ) and their average (blue line), three CoDa populations (◊) and their average (pink line). Shown are the averages of two experiments for each cell line. (b) The representative images of 53BP1 foci in the CoDa and FBCL cells at 8 and 24 hours after 2 Gy radiation treatment. DAPI staining shows the location of nuclear. In addition, double staining with 53BP1 and γH2AX antibodies was performed in FBCL and CoDa cells at various time points after 2 Gy of γ-irradiation. (c) The representative images of 53BP1 and γH2AX foci in FBCL and CoDa cells at 30 min, 8 and 24 hours after 2 Gy of radiation treatment. DAPI staining shows the location of nuclear.

The 53BP1 protein is recruited to DNA damage sites and forms nuclear foci in response to DSBs. We also measured the kinetics of 53BP1 nuclear focus formation in CoDa cells. Figure 4a shows the average number of 53BP1 nuclear foci among three CoDa cell population (CoDa-2, CoDa-21, and CoDa-33) and three normal fibroblasts (FBCL, HSF42, and HSF43). It is clear that, similar as γH2AX (Fig. 3), the CoDa cells have normal initial 53BP1 focus formation in response to ionizing radiation but a slower removal of the 53BP1 foci after IR (Fig. 4a). Significantly more 53BP1 foci can be detected in CoDa cells than control cells even at 24 hours after 2 Gy irradiation. Figure 4b shows representative images of 53BP1 foci.

We further tested if the co-localization between 53BP1 and γH2AX foci was affected in CoDa cells. Figure 4c shows the representative images for double staining of 53BP1 and γH2AX foci. More than 90% of 53BP1 and γH2AX foci were co-localized at 30 min, 8 and 24 hours after 2 Gy γ-ray treatment in both FBCL and CoDa cells. This finding suggests that the activation of DNA damage recognition pathway leading to 53BP1 and γH2AX recruitment has taken place in CoDa cells. Collectively, Figures 3 and 4 suggest a delayed repair of DSBs in CoDa cells but the DNA damage signaling to activate γH2AX, and 53BP1 recruitment are unlikely affected in CoDa cells. These data also suggest that the genetic defects in CoDa cells are distinct from Riddle's syndrome that has defect in 53BP1 recruitment due to the RNF168 dependent pathway [20], [21].

### CoDa cells have normal G1/S and G2/M checkpoints activation and ATM activation, but impaired DNA repair in response to radiation

**Figure 5:**
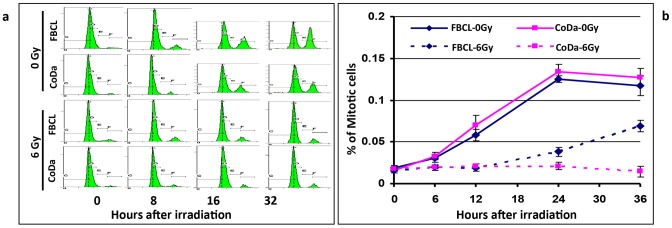
Normal G1/S and G2/M checkpoint activation and impaired recovery from G2 arrest in CoDa cells after irradiation. To assess G1/S checkpoint activation (panel a), cells were treated with or without 6 Gy γ-rays and nocodazole was added into medium right after irradiation. Cells were collected for DNA content analysis at 0, 8, 16 and 32 hours after nocodazole added. Shown in panel-a are the histographs of DNA content measured from fibroblasts with 0 Gy and 6 Gy radiation treatment. To measure G2/M checkpoint activation (panel b), cells were treated with or without 6 Gy γ-rays and nocodazole was added into medium right after radiation. Cells were fixed at 0, 6, 12, 24 and 36 hours after adding nocodazole. Then immunofluorescent staining was performed with anti-phosphorylated histone-3 (Phospho-H3 at Ser10) antibody. Percentage of mitotic cells (Phospho-H3 positive) was counted by flow cytomertry and the experiment was repeated once. Error bars are standard deviation.

Ionizing radiation has been shown to activate cell cycle checkpoints to facilitate DNA repair [22]. To determine G1/S checkpoint activation induced by IR, the cells were treated with or without 6 Gy γ-rays, and 0.5 µg/ml of nocodazole was immediately added to the cell culture after irradiation. The cells were collected at various time points and DNA content was measured by flow cytometry. Nocodazole treatment arrests cells in M phase, thus the progresses of G1 phase cells in the cell cycle can be visualized by gradual decrease of G1 population. As shown in Figure 5a (top of the panel), without irradiation, CoDa and FBCL have similar cell cycle profiles during the course of G1 phase cell population shift to G2/M phases. After 6 Gy of radiation, the there was a similar delay of the G1 shift in both CoDa and FBCL as shown in Figure 5a (bottom of the panel), indicating the irradiation induced the activation of G1/S checkpoint in both cell lines.

Histone-3 is phosphorylated at Serine-10 when the cells exit G2-phase and enter into mitosis. To test whether CoDa cells have functional G2/M checkpoint, the phosphorylated histone-3 (phosphor-H3) was stained at various time points after irradiation and nocodazole trapping. The percentages of phosphor-H3 positive cells were scored by flow cytometer. As shown in Figure 5b, there is a similar delay of mitotic index initially after 6 Gy-irradiation in both control and CoDa cells. This indicates that both cells have normal G2/M check point activation. At about 24 hours after irradiation, the G2 phase cells of normal fibroblasts start to enter into mitosis. However, the CoDa cell remains arrested at G2, event up to 32 hours after IR, suggesting a delay in recovery of G2 arrest in CoDa cells. This is consistent with the delay of DSB repair in CoDa cells (Figs. 3 and 4).

**Figure 6:**
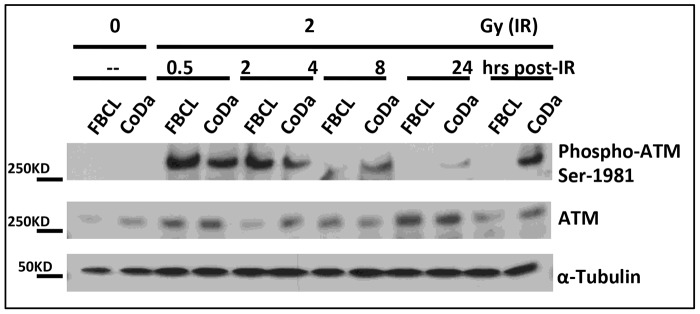
Normal activation but with delayed recovery of ATM in response to irradiation but with delayed recovery. FBCL (Control) and CoDa cells were treated with 2 Gy γ-rays and harvested at 0.5, 2, 4, 8 and 24 hours after IR for whole cell extract preparation. The protein samples were subjected to SDS-PAGE, then the membrane was blotted with antibodies specified in the figure, including ATM and phosphorylated ATM at Ser-1981, and α-tubulin was used as loading control.

The effective G1/S and G2/M checkpoint activations and the initial formation of γH2AX and 53BP1 nuclear foci after irradiation (Figs. 3, 4, and 5) suggest a normal ATM dependent signaling in CoDa cells. ATM phosphorylation at Ser-1981 is an early event after DNA damage induced by radiation. As shown in Figure 6, ATM phosphorylation was detected as early as half an hour after radiation in both control and CoDa cells. However, CoDa cells maintained a higher ATM phosphorylation level up to 24 hours compared to less than 8 hours in control cells, likely due to a high level of unrepaired DNA damage. Collectively, these data (Figures 3, 4, 5, 6) support a scenario that CoDa cells possess normal initial DNA damage response signaling, and cell cycle checkpoint activations but have an impaired repair of DSB.

### Variable Sensitivities of Dubowitz syndrome fibroblasts to different types of DNA damage agents

**Figure 7:**
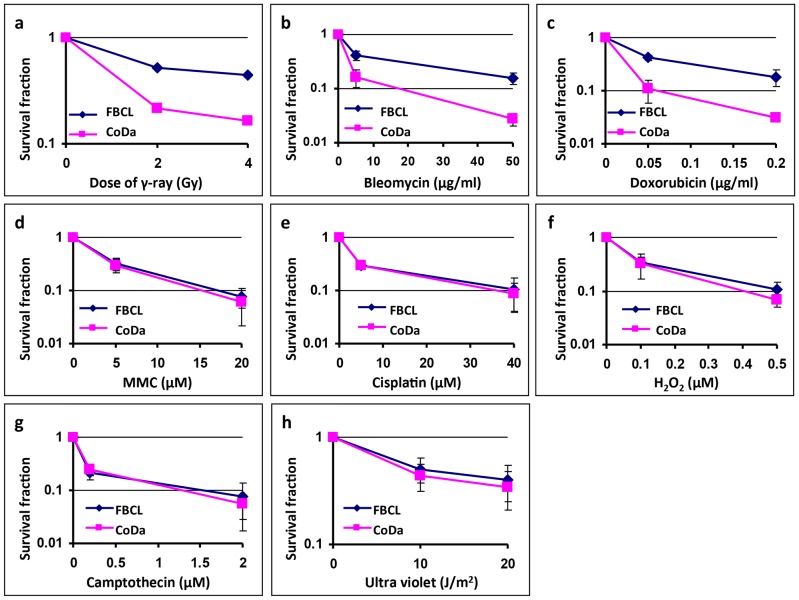
Cellular sensitivity of CoDa cells to various DNA damaging agents. The cellular sensitivity of CoDa cells was assessed using growth inhibition assay with continuous drug treatment (see Materials and Methods for details), including bleomycin (b), doxorubicin (c), mitomycin-C (d), cisplatin (e), hydroxyperoxide(f) and camptothecin(g). Panels a and h show the sensitivities with ionizing radiation and ultra violet treatment using the same growth inhibition assay as drug treatments. Shown are the averages form 3–5 independent experiments. Error bars are standard deviations.

To determine whether CoDa cells are also defective in repair of other types of DNA damages, we measured the fraction of viable CoDa cells 6 days after continuous treatments with a variety of DNA damage agents. As shown in Figure 7, CoDa cells are more sensitive to bleomycin and doxorubicin than the control cells (Fig. 7b and c), but are not to topoisomerase I poison (camptothecin) and DNA cross-link agents (cisplatin and mitomycin-C) (Fig. 7d, e and g). The sensitivity to UV and H_2_O_2_ are not significantly different between CoDa and controls (Fig. 7f and h). These data suggest that the repair defect in CoDa cells is mostly specific to DSB.

Radiation induced DSBs are mainly repaired by non-homologous end joining (NHEJ) or homologous recombination (HR). HR defects cause hypersensitivity to DNA cross-links but the CoDa cells are not sensitive to mitomycin-C and cisplatin (Fig. 7d and 7e), and we did not observe defective RAD51 recruitment to DNA damage at early stage after irradiation (Fig. S1a). Thus, we directed our attention on the major players in the NHEJ pathways. We first found normal focus formation of phosphorylated DNA-PKcs (T2609) in CoDa cells in response to irradiation (Fig. S1b).

### Identification of Mutations of genes in on Non-homologous End Joining pathway

**Figure 8:**
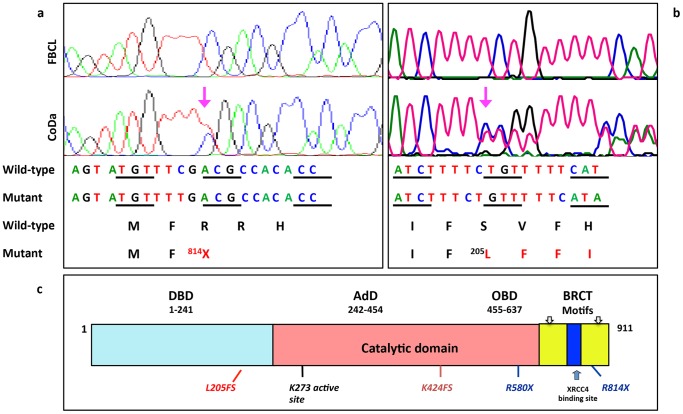
Mutations of *LIG4* identified in CoDa genomic DNA. Shown are sequencing results from control fibroblast FBCL (Wild-type) and CoDa cells (mixture of wild-type and mutated alleles). (a) The 2440C>T (R814X) mutation. Single nucleotide mutation (C>T) at position of 2440 of coding region changes Arg-814 to stop codon, which results in premature truncation of the DNA ligase IV protein. (b) The 613ΔT mutation. Single nucleotide deletion at nucleotide position 613 of coding region leads to a frame shift at L205 (L205FS) and causes a premature stop codon 18 aa downstream. Mutated amino acids are labeled with numerical number in protein sequence and shown in red. Pink arrows indicate the location of mutated nucleotides. (c) The schematic structure of DNA Ligase IV protein (Adapted from Marchetti et al. [36]). LIG4 comprises three domains: DNA-binding domain (DBD), adenylation domain (AdD) and oligo-binding domain (OBD). AdD and OBD form the catalytic domain. Two C-terminal BRCT motifs and XRCC4-binding sites (grey) are required for interaction with XRCC4. Localization of truncation mutations found in the LIG4 is labeled in the panel.

We then performed RT-PCR on CoDa cells and sequenced the cDNAs of several NHEJ genes including *KU70 (XRCC6), KU80 (XRCC5), XRCC4, DCLRE1C (Artimes), XLF (Cernunnos)* and *LIG4 (DNA Ligase IV)*. In addition, we also sequenced *P53*, *RAD51*, *CHK2*, and *BRCA1*. Two mutations in the *LIG4* gene were identified in both genomic DNA and cDNA sequencing (Fig. 8 and Fig. S2), while no mutations were observed in other sequenced genes. One mutation is a single base deletion at coding nucleotide 613 (613ΔT), which results in a reading frame shift at amino acid L205 (L205FS) and generates a new stop codon at amino acid 232 (Fig. 8b). The other is a single base mutation (2440C>T) that generates a stop codon (R814X) (Fig. 8a). To distinguish whether the two mutations occurred on the same or different copies of the chromosomes, we cloned the CoDa genomic LIG4 into a plasmid, so that each clone was derived from a single copy of chromosome. Among 14 independent clones, 7 clones contain the 613ΔT mutation without the 2440C>T mutation, and the other 7 clones only contain the 2440C>T mutation. Thus, we are confident that the two mutations are located on separate copies of chromosomes. The wild type *LIG4* was not detected in CoDa cells.

The human *LIG4* gene encodes a protein of 911 amino acids by a single long exon. As shown in Figure 8c, it contains a DNA binding domain (amino acids 1–241), and two BRCT motifs (amino acids 658–743 and 801–911) [23]. The 2440C>T (R814X) truncation mutation has been previously reported in DNA ligase IV syndrome [24]. This truncation deletes one of the C-terminal BRCT motif, significantly impairs LIG4 binding with XRCC4 and reduces its nick ligation activity by∼90% [24]. The 613ΔT (L205FS) mutation has never been reported. It codes a protein containing only part of the DNA binding domain of LIG4 and appears to be the shortest truncation of LIG4 reported so far. Two nuclear localization signals (NLS) have been identified in LIG4, located at the C-terminus of the protein near the first BRCT motif. The novel mutation we identified in *LIG4* gene, 613ΔT (L205FS), only contains part of DNA binding domain without either of NLS, and most likely will result in the nonsense-mediated decay. Two nonsense mutations (R580X and R814X) and a frame-shift (K424FS) that cause protein truncations have been reported (Fig. 8c), and all other mutations cause amino acid substitutions and deletion [23]. So far all mutations identified in LIG4 syndrome patients contain at least one allele with a hypomorphic mutation, which retains residual albeit reduced activity of the enzyme for the survival of the patients [23].

### The DNA damage sensitivity in Dubowitz syndrome cells can be complemented by expression of wild type DNA ligase IV alone

**Figure 9:**
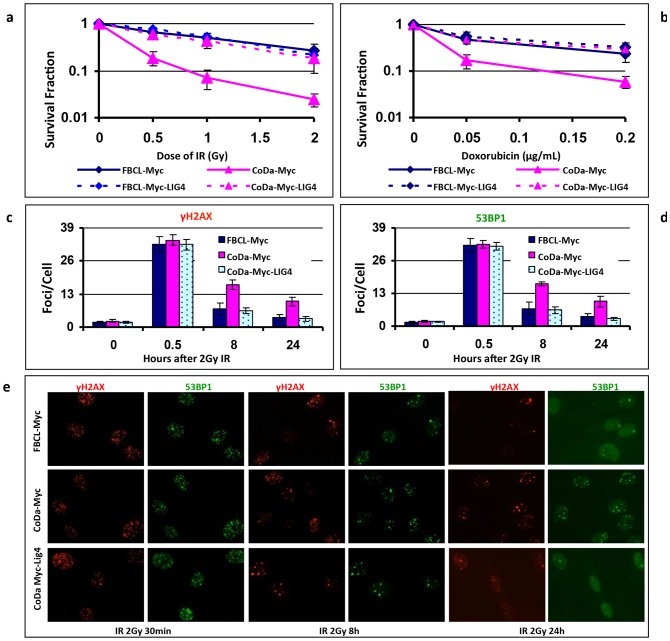
Expression of wild type LIG4 complements the cellular sensitivity of CoDa cells to DNA damage agents and rescues DSB repair defect. CoDa and FBCL cells were infected with retrovirus that express Myc-LIG4 or Myc (negative control) and selected with puromycin for 1 week. The cells were used for cellular sensitivity analysis or fluorescent immunostaining. Panel (a) shows the survival curve with irradiation treatment measured with colony formation assay. Panel (b) shows the cellular sensitivity to doxorubicin treatment assessed by growth inhibition assay (see Materilas and Method for details). For immunostaining, the cells grown on coverglass were fixed at various time points after 2 Gy IR and co-stained with anti-γH2AX (Red) and anti-53BP1 (Green) antibodies. DAPI staining (Blue) shows the location of nuclear. The numbers of γH2AX and 53BP1 foci were counted in at least 200 cells from each slide and the experiments were repeated twice. Shown are the averages nuclear focus numbers of γH2AX (Panel c) and 53BP1 (Panel d). Error bars were the standard deviations. Panel e is representative images taken at 30 min, 8 hour and 24 hours after 2 Gy irradiation.

**Figure 10:**
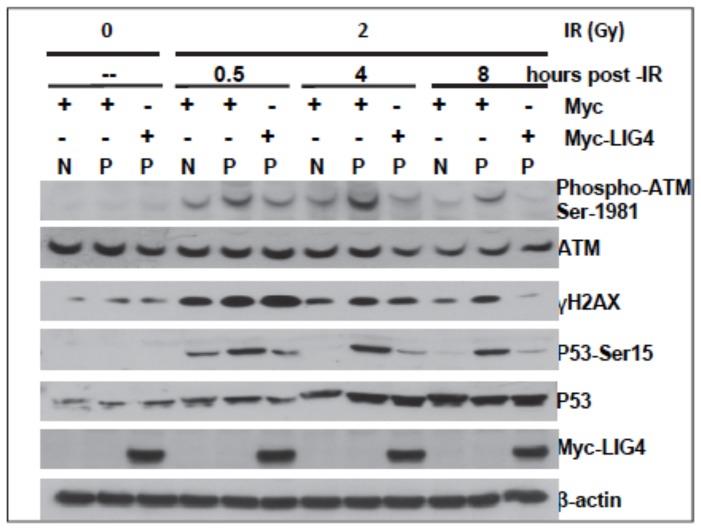
expression of LIG4 in CoDa cells does not affect the initial activation of ATM, p53, and H2AX, but eliminates the excessive residual level of phosphorylated ATM, p53, and H2AX after irradiation. After irradiation with 2 Gy of γ-rays, whole cell extracts were collected at various time points and subjected to western-blot analyses to measure the phosphorylations of ATM (Ser1981), p53 (Ser15), and H2AX (Ser139), and β-actin was used as loading control. Myc or Myc-LIG4 represents the cells were infected with retrovirus expressing Myc or My-LIG4, respectively. Protein samples from FBCL cells are labeled as “N”, from CoDa cells are labeled as “P”.

To test whether the LIG4 mutations are solely responsible for the radiation sensitivity in CoDa cells, we expressed Myc-tagged wild type LIG4 into the CoDa cells. Myc-empty vector was used as negative control. As shown in Figure 9a, expression of wild type Myc-Lig4 successfully rescued the radiation sensitivity of CoDa cells in colony formation assay. The cellular sensitivity to doxorubicin was also rescued by wild-type LIG4 as determined with growth inhibition assay (Fig. 9b). This is accompanied by reduced residual γH2AX and 53BP1 nuclear foci in the CoDa cells at later time point (8 and 24 hours) after irradiation, to a similar level as the normal fibroblasts (Fig. 9c, d, e). Furthermore, the sustained activations of ATM and p53 (due to excessive residual level of DNA damage) in CoDa cells is relieved to the same level as normal fibroblasts at 8 hours after irradiation (Fig. 10). Collectively, these data (Figs. 8, 9, 10) suggest that the CoDa cells' radiation sensitivity and defective DSB repair are solely caused by the LIG4 mutations. Thus, we have identified the genetic defects in this case of Dubowitz syndrome as compound mutations of 613ΔT (L205FS) and 2440C>T (R814X) of the human *LIG4* gene.

## Discussion

In 1965, a 13-month-old female patient was reported by Victor Dubowitz with unique clinical features [1]. Several years later, Opitz and Grosse et al. further defined the clinical features and proposed the designation of Dubowitz syndrome [2], [3]. In this report, the patient was diagnosed in 1973 at one-year-old age, and she was one of the cases used to define the common clinical features of Dubowitz Syndrome [3].

We established fibroblast cell lines from this patient and the availability of the cell lines provides us with the opportunity to characterize the DNA repair defects and to identify its genetic defects. This is the first time to our best knowledge, that the genetic defects of Dubowitz syndromes are identified, despite the fact that Dubowitz syndrome has been originally reported in 1965. Among Dubowitz syndrome cases described in literature, few of them were evaluated on molecular level. A case of high frequency of chromosome breaks in the lymphocytes of two sister-and-brother siblings with Dubowitz Syndrome after nitrogen mustard and diepoxybutane treatments was reported in 1991 [9]. Another report mentioned a high rate of chromosome breakage in a case of Dubowitz syndrome with embryonic rhabdomyosarcoma [11]. Although those reports have implicated DNA repair defects in Dubowitz Syndrome, it is until now that the direct evidence of DNA repair defects is found.

The knowledge that *LIG4* mutation is responsible for the disease should have significant clinical impact for the care of similar Dubowitz syndrome patients. Because CoDa cells from our patient are sensitive to ionizing radiation, bleomycin, and topoisomerase II poison, but not sensitive to DNA cross-link agents or topoisomerase I poison. Thus, DNA crosslinking agents such as mitomycin C and cisplatin, or Topo I poisons camptothecin and topotecan, should be the preferred choices for those patients with cancer. Ionizing radiation, bleomycin and doxorubicin should be avoided to reduce the severe side effect. In addition, our finding also offers molecular bases to manage the immunological defects as reported in some of the Dubowitz syndrome cases [7], [10].

Dubowitz syndrome was described in 1965 [1]. It has been implied that Dubowitz syndrome may have some clinical overlap with other DNA repair defective inheritable diseases, including Bloom's syndrome and DNA ligase IV syndrome [25]. DNA ligase IV mutation was firstly reported in a radiation sensitive leukemia patient [26], and DNA ligase IV syndrome was soon formally proposed in 2001 [24]. However, ligase IV syndrome is a disorder genetically defined by the mutations on the *Ligase IV* gene. On the other hand, Dubowitz syndrome is a diagnosis based on clinical features, but not by genetics. The term of “Dubowitz syndrome” was coined many years before Ligase IV syndrome was introduced into the scientific field. Our study suggested that some of the Dubowitz syndrome patients diagnosed prior to the awareness of Ligase IV syndrome might be contributed by Ligase IV mutations genetically, and that a subset of Dubowiz syndrome is the same as DNA ligase IV syndrome. This finding puts Dubowitz syndrome and DNA ligase IV syndrome into the same molecular group for future diagnosis and treatment planning.

## Materials and Methods

### Establishment and characterization of CoDa cells

The human subject issues of this work have been reviewed and approved by Institutional Review Board of Robert Wood Johnson Medical School. It was determined that the use of cell lines established from autopsy is exempt from the human subject.

Skin tissue blocks were obtained from autopsy of the patient described in Result. The skin blocks were washed with PBS in a Petri dish and cut into small cubic pieces (1–2 mm^3^) before transferred to a 6-well plate. In each well, 7–10 small tissue fragments were evenly distributed over the culture surface. The tissue blocks were incubated in 37°C incubator with 5% CO_2_ for 4 hours to allow tissue pieces to be settled. Then, 1 ml of complete growth medium was carefully added into each well without disturbing the tissue blocks. Complete growth medium is composed of α-MEM cell culture medium (Sigma) supplemented with 10% fetal bovine serum (FBS, Sigma) and 0.2 mM glutamine, 50 U/ml penicillin, 50 mg/ml streptomycin. The medium was replaced after 5 days and replaced every 3 days thereafter. When the fibroblasts grown from the tissue fragments were nearly confluent, the tissue blocks were removed and the cells were trypsinized and re-plated into 6-cm Petri dishes, which was designated as passage 1. The established primary fibroblasts were named as CoDa, the initials of the patient. Several independent populations of fibroblast lines (CoDa-2, CoDa-25, and CoDa-33) were established from independent wells of culture plates. Passaging and subculture of CoDa cells were performed with 1∶3 dilution when the cells reached ∼90% confluence.

### Determinations of cell population doubling time and plating efficiency

To determine the population doubling time of the cells, 1.8×10^5^ viable log-phase cells were seeded into 60 mm culture dish at day 0. After 24 hours of incubation (Day 1), 3 dishes of cells were trypsinized and cell number was counted. The average cell number from 3 dishes was recorded as N1. At day 3, the average cell number from another 3 dishes was determined and recorded as N2. The following formula was used to calculate doubling time (Td): Td = T*log2/log(N2/N1), where T is the time between two counting. To determine the plate efficiency (PE), 300 log-phase cells were seeded into 100 mm culture dishes and medium was changed every 4–5 days. After 15 days of culture, the number of colonies was counted in culture dish after stained with 1% crystal violate in absolute methanol. PE was calculated as the percentage of average colony number over total number of cells seeded. Doubling time and PE were measured every 3–4 passages.

### Radiation Survival Assay with colony formation

Log-phase cells were plated on 100 mm culture dishes. The cells were irradiated with Cs-137 γ-rays (dose rate: 1.026 Gy/min) 18 hours after the cells were plated. Colonies were grown for 15 days, after which the colonies were stained with 1% crystal violet in methanol. The number of colonies was normalized to the number of cells plated to calculate the surviving fraction. Each experiment was performed in triplicate and repeated at least twice.

### Drug sensitivity measured with growth inhibition assay

A collection of 1×10^5^ viable log-phase cells were seeded into 60 mm culture dishes. Twenty-four hours later, various concentrations of drug were added to cell culture. The cells were fed with fresh medium with drug 3 days later. After total of 6 days drug treatment, the cells were trypsinized and the number of viable cells was counted. Surviving fraction was calculated by normalizing the number of survived cells to that of control cells. Each experiment was performed in triplicate and repeated at least twice.

### Immunofluorescence staining

Cells (2.5×10^4^) were plated and grown on glass coverslips for 24 hours, then treated or not with γ-rays. The procedures to stain for γH2AX and RAD51 foci have been described previously [27]–[29]. 53BP1 and phosphorylated DNA-PKcs foci were visualized by the same procedure except that anti-53BP1 (1∶1,000) primary antibody (Bethyl, Montgomery, TX) and anti-Phospho-DNA-PKcs (T2609) (1∶1,000) primary antibodies (Abcam, Cambridge, MA) were used. To visualize the expression of Myc-LIG4, anit-Myc (1∶300) primary antibody (Santa Cruz biotechnology inc., Santa Cruz, CA) was used. To detect mitotic cells, anit-phospho-Histone H3 (Ser10) primary antibody (Cell signaling technology, Danvers, MA) was used to stain the cells. Immunofluorescent signals were recorded using a Carl Zeiss fluorescent microscope (Axiovert-200M) equipped with Carl Zeiss digital camera (AxioCam MRC).

### Analyses of DNA content and mitotic index by flow cytometry

Cells were trypsinized and fixed with 70% ice-cold ethanol. DNA content was measured using flow cytometry as described before [30] and 20,000 cells were collected in each assay. The cells were treated or not with 6 Gy of γ-radiation, and 0.5 µg/ml nocodazole were added into cell culture right after irradiation for continuous treatment. At various time points after DNA damage treatment, cells were fixed for immunofluorescent staining. Mitotic (histone-3 phosphorylation at Ser10 positive) cells were counted with flow cytometer. For each sample, the percentage of mitotic cells was determined from about 10,000 cells counted, and the experiment was repeated once.

### mRNA and genomic DNA sequencing

To sequence the coding region of candidate genes, specific primers were designed based on the mRNA or genomic sequence from NCBI database. PCRs were performed with templates from CoDa cell, including genomic DNA or cDNA synthesized from total RNA extracted from CoDa cells. DNA fragments with correct size were purified for sequencing. The primer sequences are shown in Supplementary Table S1.

### Complimentary assay with DNA Ligase IV

Full length DNA Ligase IV clone was purchased from Addgene (Addgene plasmid 13332) as described previously [31]. EcoRI and XhoI sites were used to insert Ligase IV coding sequence into pLXSP-Myc retroviral vector [32], which was modified from retroviral vector of pLXSN (Clontech Laboratories Inc., Mountain View, CA) to generate N-terminal fused Myc-LIG4 (pLXSP-Myc-LIG4). The empty or pLXSP-Myc-LIG4 retroviral vector was transfected into Phoenix packaging cells to generate virus soup. The fibroblasts were infected with retrovirus and followed by the selection with puromycin (0.9 µg/ml) for 7 days. The functional assays were performed with transduced cells.

### Western blot

The cell lysate were prepared as described before [33], [34]. The samples were subjected to SDS-PAGE. The phosphorylation of ATM and P53 were detected with anti-Phospo-ATM (Ser1981) and anti-Phospo-P53 (Ser15) antibodies (Cell signaling technology, Danvers, MA). Total proteins of ATM and P53 were blotted with anti-ATM and anit-P53 antibodies (Santa Cruz Biotechnology Inc., Santa Cruz, CA). The expression of Myc-LIG4 fusion protein was confirmed with anti-Myc antibody (Santa Cruz Biotechnology Inc., Santa Cruz, CA). The levels of **γ**H2AX and RAD51 were measured with anti- γH2AX (Ser139) antibody (Upstate Biotechnology, Lake Placid, NY) and anti-RAD51 antibody (Santa Cruz biotechnology inc., Santa Cruz, CA). The level of β-actin or α-tubulin was detected with anti-β-actin or anti-α-tubulin antibody (Sigma-Aldrich, St. Louis, MO) to serve as a loading control.

## Supporting Information

Figure S1 **CoDa cells have normal RAD51and phosphorylated DNA-PKcs focus formation.** RAD51 focus formation in response to DNA damage is a hallmark of active HR machinery. Failure or delay in forming RAD51 foci would signal an impaired HR process. To integrate whether the homologous recombinational repair pathway is affected in CoDa cells, we performed immunostaining of RAD51 foci in CoDa cells. At various time points after the cells were treated with 2 Gy γ-radiation, cells were fixed and stained by immunoflurescent techniques with an anti-RAD51 antibody. The number of RAD51 foci was counted in >200 cells per time point in each experiment. Panel (a) shows the numbers of RAD51 foci per cell in FBCL and CoDa cells. Data shown are averages and standard deviations of three experiments. As shown in panel S1a, similar numbers of RAD51 foci were formed in both control and CoDa cells and reached the peak at about 4 hours after IR. However, the removal of RAD51 foci in CoDa cell is slower in CoDa than that in FBCL cells. There was no change of RAD51 protein levels in CoDa cells compared with that in control cells (Data not shown). In addition, the level of RNF8, an ubiquitin E3 ligase involved in repair proteins recruitment and the FANCD2 mono-ubiquitylation was not affected in CoDa cells (Data not shown). These data suggest that HR pathway is not likely affected in CoDa cells. Panel (b) are representative images of phosphorylated DNA-PKcs staining. The cells were fixed 1 hour after 2 Gy IR treatment and processed for immunostaining with anti-phosphorylated DNA-PKcs (T2609) antibody. Cells were treated with 0.5% triton X-100 for 5 min on ice, then fixed with 4% PPBS. Shown in panel b, the green channel is for phosphorylated DNA-PKcs and DAPI staining (Blue) represents the location of nuclear. Similar number of foci was observed in CoDa and control cells. This result indicates normal early events of NHEJ, and further suggests the normal ATM signaling in CoDa cells, which is consistent with the phosphorylation of DNA-PKcs at T2609 [35].
(TIF)

Figure S2 **Mutations of** ***LIG4*** **identified in CoDa cell mRNA.** Shown in figure are sequencing results of LIG4 cDNA ampliifed from control fibroblast FBCL (Wild-type) and CoDa cells. Panel (a) shows the nonsense mutation of 2440C>T (R814X). Panel (b) shows the frame shift mutation of 613ΔT (L205FS). Mutated amino acids are labeled with numerical number in protein sequence and shown in red. Pink arrows indicate the location of mutated nucleotides.
(TIF)

Table S1 **Primers used for LIG4 fragments amplification.** Primers labeled as genomic and cDNA are designated for genomic DNA and cDNA fragments amplification, respectively. All other primers are common for LIG4 fragments amplification.
(TIF)
