# Supplementary figures and images for: Identification of the DNA Repair Defects in a Case of Dubowitz Syndrome
Source: PLoS One. 2013 Jan 25;8(1):e54389. doi: 10.1371/journal.pone.0054389 (PMC3556036; doi:10.1371/journal.pone.0054389)

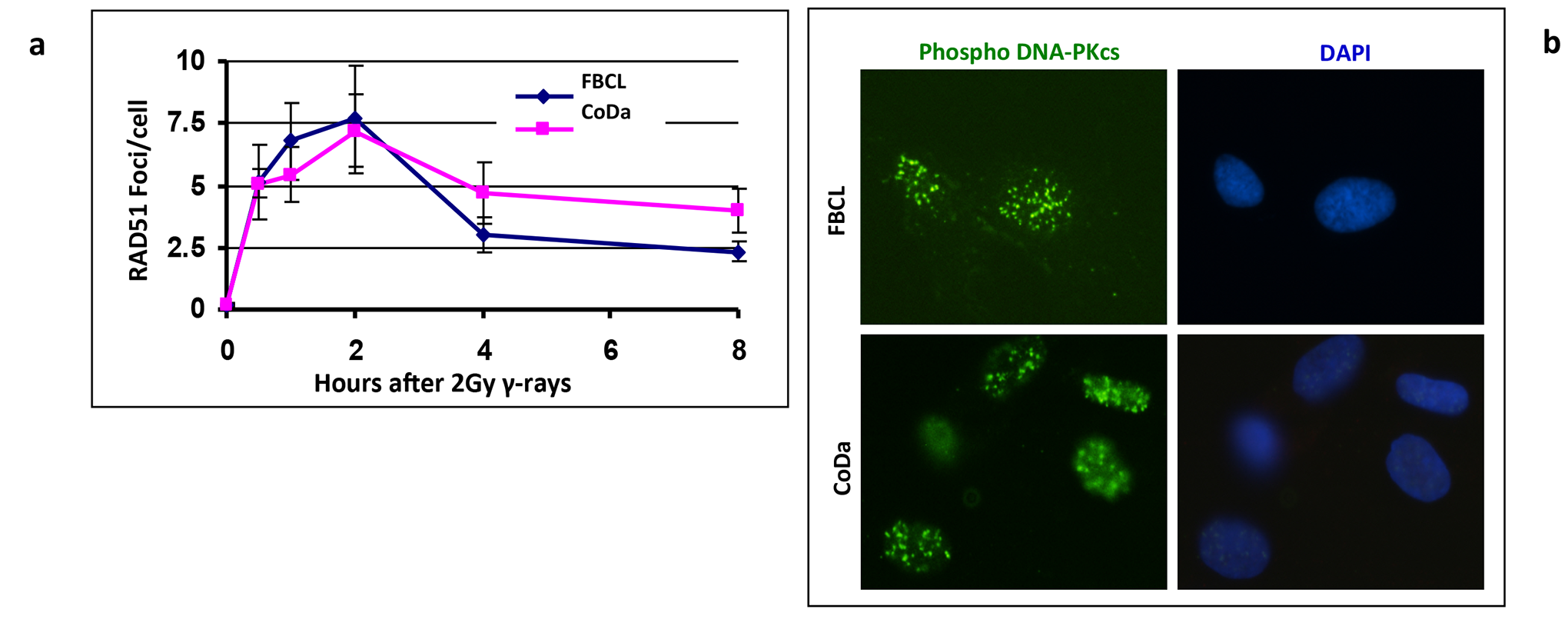

Supplement: Figure S1 — CoDa cells have normal RAD51and phosphorylated DNA-PKcs focus formation. RAD51 focus formation in response to DNA damage is a hallmark of active HR machinery. Failure or delay in forming RAD51 foci would signal an impaired HR process. To integrate whether the homologous recombinational repair pathway is affected in CoDa cells, we performed immunostaining of RAD51 foci in CoDa cells. At various time points after the cells were treated with 2 Gy γ-radiation, cells were fixed and stained by immunoflurescent techniques with an anti-RAD51 antibody. The number of RAD51 foci was counted in >200 cells per time point in each experiment. Panel (a) shows the numbers of RAD51 foci per cell in FBCL and CoDa cells. Data shown are averages and standard deviations of three experiments. As shown in panel S1a, similar numbers of RAD51 foci were formed in both control and CoDa cells and reached the peak at about 4 hours after IR. However, the removal of RAD51 foci in CoDa cell is slower in CoDa than that in FBCL cells. There was no change of RAD51 protein levels in CoDa cells compared with that in control cells (Data not shown). In addition, the level of RNF8, an ubiquitin E3 ligase involved in repair proteins recruitment and the FANCD2 mono-ubiquitylation was not affected in CoDa cells (Data not shown). These data suggest that HR pathway is not likely affected in CoDa cells. Panel (b) are representative images of phosphorylated DNA-PKcs staining. The cells were fixed 1 hour after 2 Gy IR treatment and processed for immunostaining with anti-phosphorylated DNA-PKcs (T2609) antibody. Cells were treated with 0.5% triton X-100 for 5 min on ice, then fixed with 4% PPBS. Shown in panel b, the green channel is for phosphorylated DNA-PKcs and DAPI staining (Blue) represents the location of nuclear. Similar number of foci was observed in CoDa and control cells. This result indicates normal early events of NHEJ, and further suggests the normal ATM signaling in CoDa cells, which is consistent wi [file pone.0054389.s001.tif]

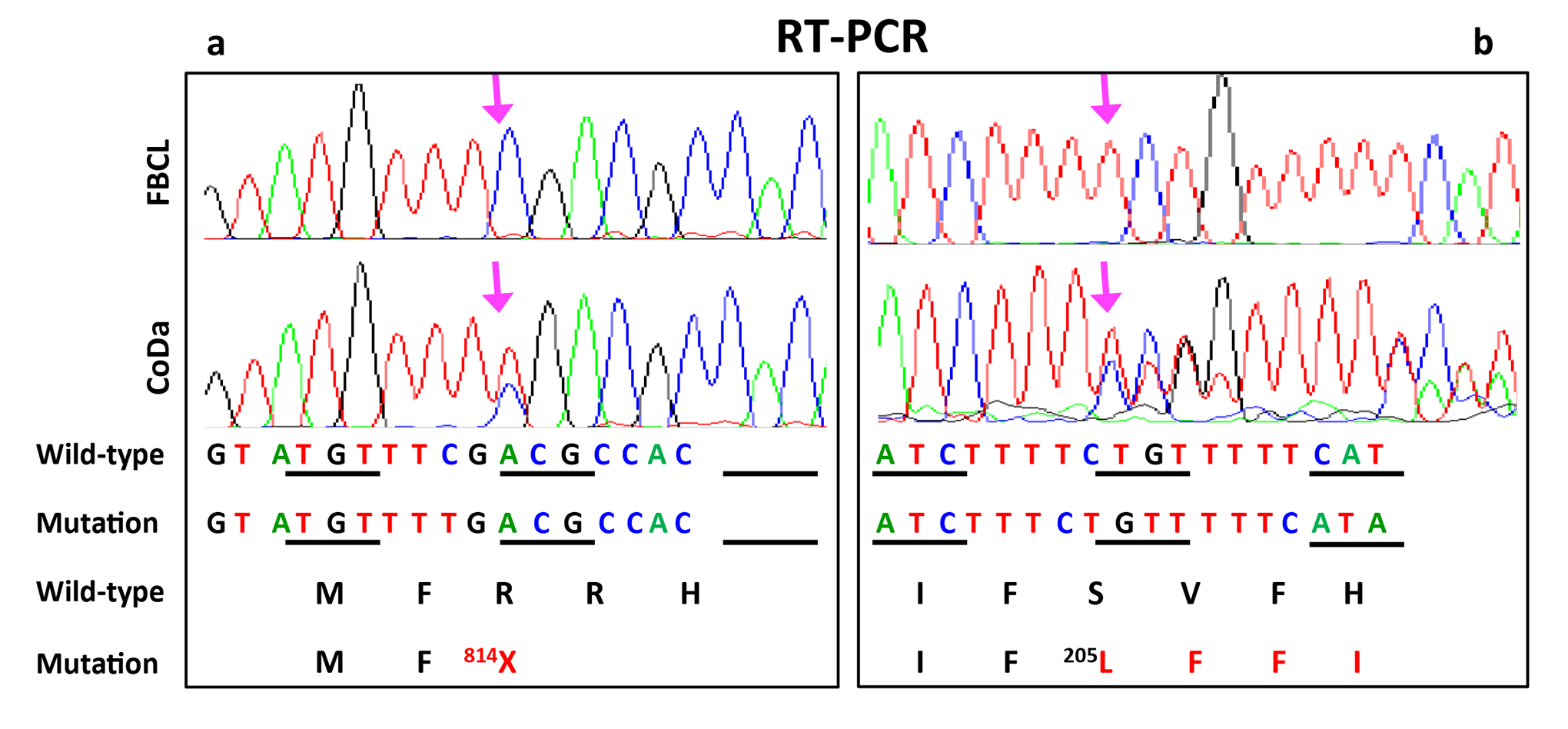

Supplement: Figure S2 — Mutations of LIG4 identified in CoDa cell mRNA. Shown in figure are sequencing results of LIG4 cDNA ampliifed from control fibroblast FBCL (Wild-type) and CoDa cells. Panel (a) shows the nonsense mutation of 2440C>T (R814X). Panel (b) shows the frame shift mutation of 613ΔT (L205FS). Mutated amino acids are labeled with numerical number in protein sequence and shown in red. Pink arrows indicate the location of mutated nucleotides. (TIF) [file pone.0054389.s002.tif]

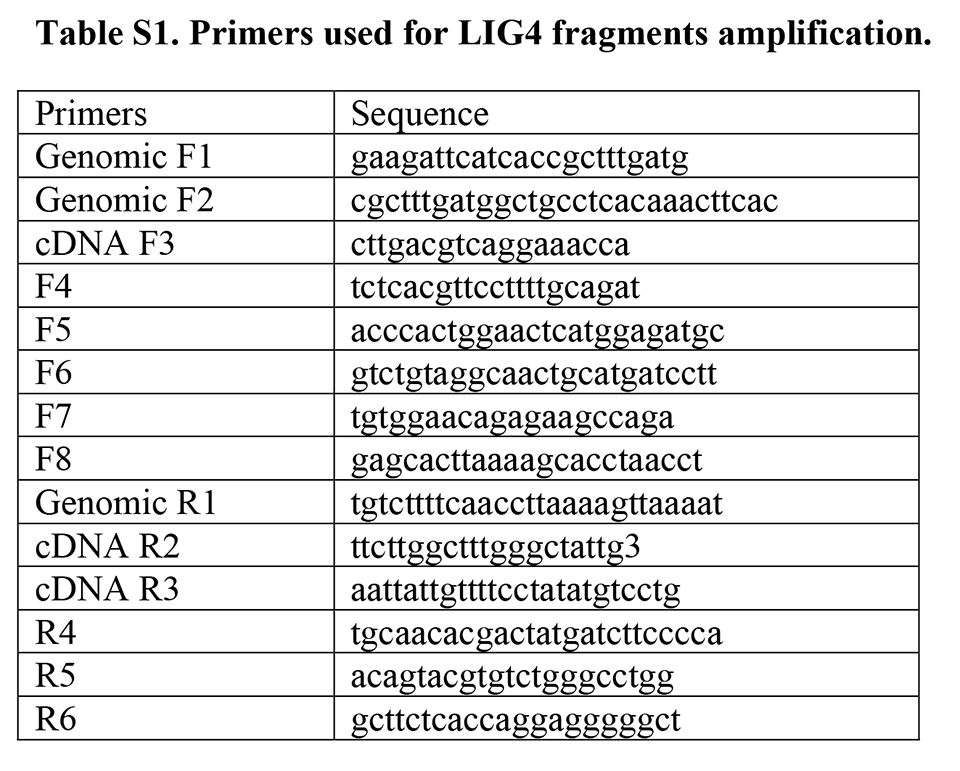

Supplement: Table S1 — Primers used for LIG4 fragments amplification. Primers labeled as genomic and cDNA are designated for genomic DNA and cDNA fragments amplification, respectively. All other primers are common for LIG4 fragments amplification. (TIF) [file pone.0054389.s003.tif]
